# Immunohistochemical Study of ASC Expression and Distribution in the Hippocampus of an Aged Murine Model of Alzheimer’s Disease

**DOI:** 10.3390/ijms22168697

**Published:** 2021-08-13

**Authors:** Diana Reimers, Manuela Vallejo-Muñoz, María José Casarejos, Adriano Jimenez-Escrig, Rafael Gonzalo-Gobernado, Eulalia Bazan

**Affiliations:** 1 Servicio de Neurobiología, Instituto Ramón y Cajal de Investigación Sanitaria (IRYCIS), 28034 Madrid, Spain; manuela.vmqm@gmail.com (M.V.-M.); m.jose.casarejos@hrc.es (M.J.C.); adriano.jimenez@hrc.es (A.J.-E.); rd.gonzalo@cnb.csic.es (R.G.-G.); eulalia.bazan@hrc.es (E.B.); 2 Servicio de Neurología, Hospital Ramón y Cajal, 28034 Madrid, Spain; 3 National Center for Biotechnology (CNB), Consejo Superior de Investigaciones Científicas CSIC, 28049 Madrid, Spain

**Keywords:** Alzheimer’s disease, neuroinflammation, innate immunity, ASC, inflammasomes, amyloid β plaques, microglia, astroglia, hippocampal interneurons, age

## Abstract

Neuroinflammation is involved in the pathogenesis of neurodegenerative diseases such as Alzheimer’s disease (AD), and is notably dependent on age. One important inflammatory pathway exerted by innate immune cells of the nervous system in response to danger signals is mediated by inflammasomes (IF) and leads to the generation of potent pro-inflammatory cytokines. The protein “apoptosis-associated speck-like protein containing a caspase recruitment domain” (ASC) modulates IF activation but has also other functions which are crucial in AD. We intended to characterize immunohistochemically ASC and pattern recognition receptors (PRR) of IF in the hippocampus (HP) of the transgenic mouse model Tg2576 (APP), in which amyloid-beta (Aβ) pathology is directly dependent on age. We show in old-aged APP a significant amount of ASC in microglia and astrocytes associated withAβ plaques, in the absence of PRR described by others in glial cells. In addition, APP developed foci with clusters of extracellular ASC granules not spatiallyrelated to Aβ plaques, which density correlated with the advanced age of mice and AD development. Clusters were associated withspecific astrocytes characterized by their enlarged ring-shaped process terminals, ASC content, and frequent perivascular location. Their possible implication in ASC clearance and propagation of inflammation is discussed.

## 1. Introduction

Inflammation is an important process in the pathogenesis of neurodegenerative diseases such as AD, involving activation of both microglia and astrocytes. Activated microglia includes a range of phenotypes under conditions of chronic inflammation, in which the pro-inflammatory M1 phenotype and the anti-inflammatory, “alternatively activated” M2 phenotype represent the opposite extremes of this spectrum [1,2]. The former is characterized by the expression of pro-inflammatory cytokines (IL-1β, IL-6, IL-12, IL-18, and tumor necrosis growth factor α (TNFα)), radical oxygen species (ROS) and nitric oxide synthase 2 (NOS2), and by impaired phagocytic activity, while the latter phenotype is implicated in phagocytosis and Aβ clearance, and secretion of anti-inflammatory cytokines as IL-4, IL-10, IL-13, and TGF-β [2,3,4,5]. In the same line, in the case of astroglia, some authors refer that this cell type is also able to switch from metabolic support cells to immunological cells, capable of producing pro-inflammatory factors [6,7]. Of great interest are several reports indicating that microglial dysfunction in long-term AD may be reversible and that thephagocytic activity of these cells may be modulated to reduce amyloid accumulation [8,9,10].

Recent studies have uncovered that microglia, and in some cases also astrocytes, have the intrinsic ability to respond as innate immune cells to danger signals through the activation of multiprotein complexes termed inflammasomes (IF) [5,11]. The molecule ASC is an essential mediator of this activation, which final purpose is to generate potent pro-inflammatory cytokines, i.e., IL-1β, IL-18, and IL-33. In these complexes, ASC bridges activated cytosolic pattern recognition receptors (PRR) sensing danger signals to the effector protein pro-caspase-1. This results in caspase-1 activation, which then cleaves the pro-interleukins intotheir active counterparts. Subsequently, the cells release IL-1β, which is a key initiator of inflammation when sensed by its widely expressed receptor, triggering NF-κB-dependent transcriptional events and further cytokine production [4,11,12]. The cytosolic PRR is frequently a member of the “Nucleotide-binding and Oligomerization Domain (NOD)-like Receptor (NLR) family, which contains either an N-terminal pyrin domain (PYD) (NLR family PYD containing 3: NLRP3, among others), or an N-terminal caspase recruitment domain (CARD) (NLRC4 and NLRP1, among others). ASC itself is also composed of a CARD, a PYD, and a linker between both domains [13].

Many recent studies have implicated ASC-containing IF in the development of AD [5,12,14]. FibrillarAβ is able to induce in microglial cells primed with lipopolysaccharide (LPS) the activation of NLRP3-ASC IF and of caspase-1, leading to the maturation of IL-1β [1,15]. In an AD murine model, the down-regulation of this IF significantly increases microglial phagocytic activity and the expression of the proteolytic insulin-degrading enzyme, thereby increasing Aβ clearance, reducing its load, and ameliorating cognitive impairment [1]. In the case of rat primary astrocytes, danger signals as palmitate are able to induce the activation of NLRC4-ASC IF [16]. The down-regulation of this PRR reduces the generation of Aβ levels produced by primary neurons cultured in conditioned media from palmitate-treated astrocytes [17].Studies conducted in ASC knockout mice reveal that the levels of the component of IF ASC are inversely correlated with the phagocytic activity of glial cells and of the amyloid load [18].

As a physiological cellular response to inflammasome activation, ASC in macrophages and microglia oligomerizes and forms micrometer-sized aggregates termed ASC specks, which are required for efficient IL-1β processing [19,20,21]. In fact, these specks, which possibly function as a signal amplification mechanism for caspase-1 activation [22], when released to the extracellular space due to inflammatory cell death, retain the ability to mature pro-IL-1β. Thus, they are sensed as endogenous danger signals and are phagocytosed by neighboring macrophages, which in turn produce IL-1β and release more ASC specks. This sequence of events may be a mechanism of inflammation transmission from cell to cell, resulting in the perpetuation of inflammation in a largely sensor-independent way [23].

It is important to highlight that ASC is able to regulate inflammation and IL-1β processing independently of the classical caspase-1containing IF. In fact, the protein rather promotes cell-mediated responses in antigen-induced arthritis, and ininfluenza virus infection [24]. Furthermore, ASC has been implicated in other processes different frominflammation. ASC, which is silenced by DNA methylation in many different types of tumors, acts as a tumor-suppressor agent [25]. The pro-apoptotic mechanism of the protein is mediated by caspase-9, or caspase-8 and Bax [24,26]. ASC is also able to trigger another type of cell death after stimulation of macrophages with pro-inflammatory stimuli as LPS, termed pyroptosis, which is independent of the influence of IF [27,28]. This cell death, which is characterized by osmotic lysis, the disintegration of the cell membrane, and passive release of the intracellular pro-inflammatory cytokines, is mediated by the formation of pyroptosomes. These structures are large supramolecular assemblies composed of oligomerized ASC dimers, which rapidly activate caspase-1 [28].

More relevant tothe present study is an important recent finding in AD patients and AD murine models, which demonstrates that ASC specks released by microglia bind rapidly to Aβand increase the formation of Aβ oligomers and aggregates. As a consequence, extracellular ASC specks act as an inflammation-driven cross-seed, which promotes Aβ pathology spreading [21].

There are still few studies analyzing in vivo ASC expression in AD models of aged mice. AD is an age-related neurodegenerative disease, and, in the same way, neuroinflammation is notably dependent on age [4,5]. IL-1β is increased in the central nervous system of mice and rats with age, and fibrillar Aβ induces significantly more IL-1β in microglia prepared from aged versus young mice [4]. Thus, age-related microglial and possibly also astrocytic changes have the potential to promote AD pathology, suggesting that age influences AD progression via modulation of glial immune responses [5]. The Tg2576 transgenic mouse line is especially interesting for analyzing the influence of age in Aβ pathology since the development of this pathology is directly dependent on age in these mice. At 11–13 months of age, their brains show a five-fold increase in Aβ_1-40_ and a fourteen-fold increase in Aβ_1-42_, in addition to initial Aβ plaques which continue spreading, so that in elderly animals the plaque load is far greater and more widespread [29,30]. Aβ deposits in thesemice begin in the cortex and HP but then spread throughout the brain [31].

In the present study, our aim was to analyze the expression and distribution of ASC in the HP of 20 to 22 month-old Tg2576 transgenic mice. We find herein that, in these old-aged mice, microglia, and several astrocytes surrounding Aβ plaques in the HP, expressed ASC in their soma and in most prolongations in contact with plaques. Microglia also expressed the protein in their nuclei. Apparently, in these locations, this protein was not associated either with NLRP3, NLRP1, or NLRC4. In addition, we reveal along several hippocampal strata, a not previously reported distribution of ASC in extracellular granules grouped in different foci, each of which contains one specific hippocampal astrocyte expressing cytoplasmic ASC and intimately associated with these granules. In order to analyze some aspects of the function of ASC in these locations, we give a detailed description of the relation of ASC with surrounding cells and tissue components.

## 2. Results

**Figure 1:**
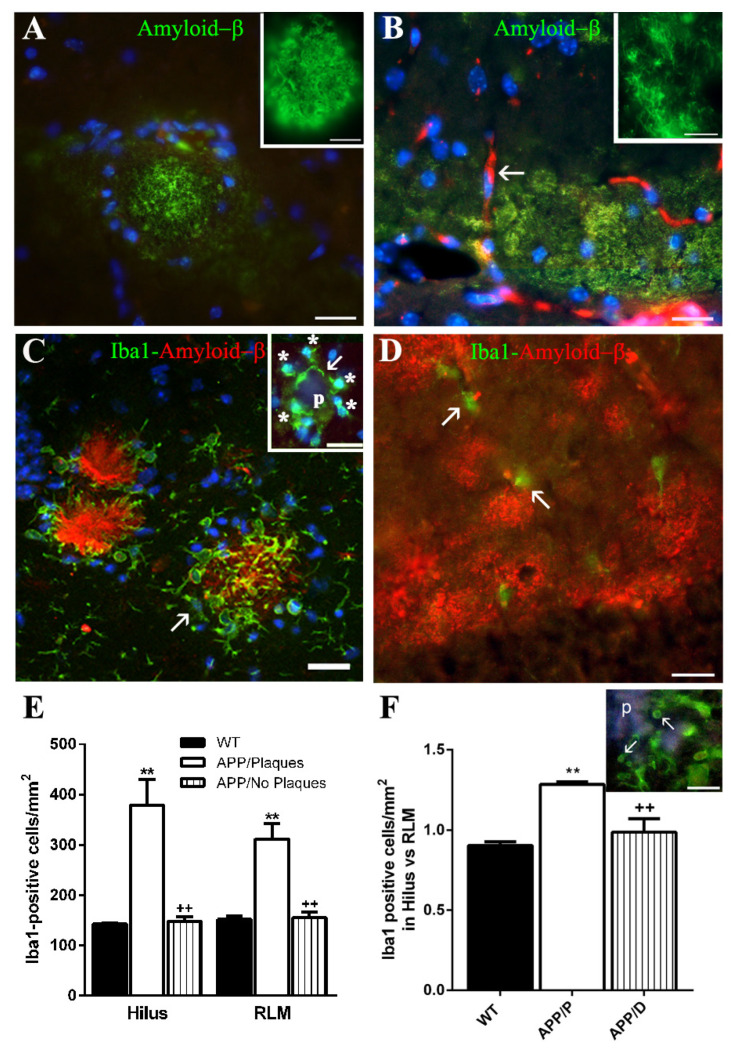
Microglia response to Aβ depends on the type of amyloid deposit to which they associate. (**A**,**B**) show the morphological differences between round and condensed Aβ plaques ((**A**) and inset) and diffuse, fibrillar, and irregularly shaped Aβ deposits ((**B**)and inset) in the HP of 22-month-old APP. Note that cell nuclei disposition (stained with Hoechst in blue) is also different, since they surround the plaque in (**A**), but are dispersed in the diffuse deposits in (**B**).The arrow in (**B**) indicates the presence of erythrocytes (red autofluorescence) since this brain was not perfusion-fixed. (**C**) Confocal image showing the distribution of activated microglial Iba1-positive soma and processes surrounding or even penetrating (arrow) the plaques, which is rather different from the dispersed and not activated microglia in diffuse deposits shown in (**D**) (arrows). Note how the Iba1-positive processes (arrow) of five microglial cells (asterisks) embrace a small plaque (p) in (**C**), inset, and the Iba1-positive process loops indicative of phagocytosis in (**F**), inset (arrows). The graph in (**E**) shows that the density of microglial cells in APP/P is ~2.7 fold higher than in APP/D and WT.The graph in (**F**) reveals that the quotient between microglial density in hilus versus strata RLM is significantly higher (>1) in APP/P than in WT and APP/D. Results represent the mean ± SEM of 3 to 4 independent mice per experimental group. A one-way ANOVA followed by the Newman–Keuls test was performed. ** *p* ≤ 0.01 vs. WT. ++ *p* ≤ 0.01 vs. APP/P. Scale bars in (**A**,**B**,**D**): 20 µm and in insets: 5 µm; in C: 25 µm and inset: 20 µm; in F inset: 10 µm.

All APP mice herein analyzed expressed the APP695 Swedish transgene in their genotype. Of the 27 APP mice which were fixed by perfusion, 26% developed a significant number of Aβ plaques (HP with ≥ 10 plaques/mm^2^) with a condensed appearance (Figure 1A and inset). This animal group, named herein APP/P, (n = 7), developed in their HP at 22 months of age 34.88 ± 6.65 plaques/mm^2^, with a round and condensed appearance, as well as diffuse, irregularly shaped Aβ deposits with a lax fibrillar structure. The remaining (74%) of APP mice, APP/D (n = 20), showed few or none Aβ plaques (<10 plaques/mm^2^), but developed instead diffuseAβ deposits, especially in the stratum lacunosum-molecularis of the HP and in the stratum molecularis of the dentate gyrus (DG). The presence of these deposits in APP animals thatdid not develop plaques was confirmed in immersion-fixed APP brains, where the fibrillar nature of diffuse deposits was better visualized (Figure 1B and inset).

### 2.1. Amyloid-β Plaques Were Surrounded and/or Traversed by Processes Which Expressed ASC, Arising Mainly from Microglia but also from Astrocytes. PRR (NLRP1,NLRP3 and NLRC4) Indicative of Inflammasomes Were Not Detected in Old-Aged APP

Microglia surrounding the plaques frequently polarized their processes, which could be oriented perpendicularly towards the plaques or actually penetrated into the plaques, as shown in confocal optical sections (Figure 1C, arrow) of z-stacks, exhibiting loops indicativeof phagocytic activity (Figure 1F inset, arrows). In other plaques, microglial processes showed few or no polarization, but in some cases, they embraced the plaques (p) (Figure 1C inset, arrow), apparently encapsulating and separating them from the surrounding tissue. By contrast, microglia immersed in diffuse deposits had a dispersed distribution and apparently did not react to the surrounding Aβ (Figure 1D, arrows).

At this point, we analyzed if microglial cells associated with diffuse Aβ deposits did not respond with activation and proliferation in APP/D, while those related with plaques did so in APP/P. Therefore, we quantified the number of microglial cells (Iba-1 positive) per mm^2^ in the HP of the three experimental groups (3 WT, 4 APP/P, and 3 APP/D). As shown in Figure 1E, the density of microglial cells in hilus and in the strata radiatum, lacunosum-molecularis of HP and molecularis of DG (RLM) in APP/P was significantly higher than that of WT and APP/D, which were similar to each other. As seen in Figure 1F, the proliferation rate of microglia was increased in hilus versus RLM, since the ratio between microglial density in the former and in the latter was >1 in APP/P and significantly higher than in WT and APP/D, where it was around 1.

**Figure 2:**
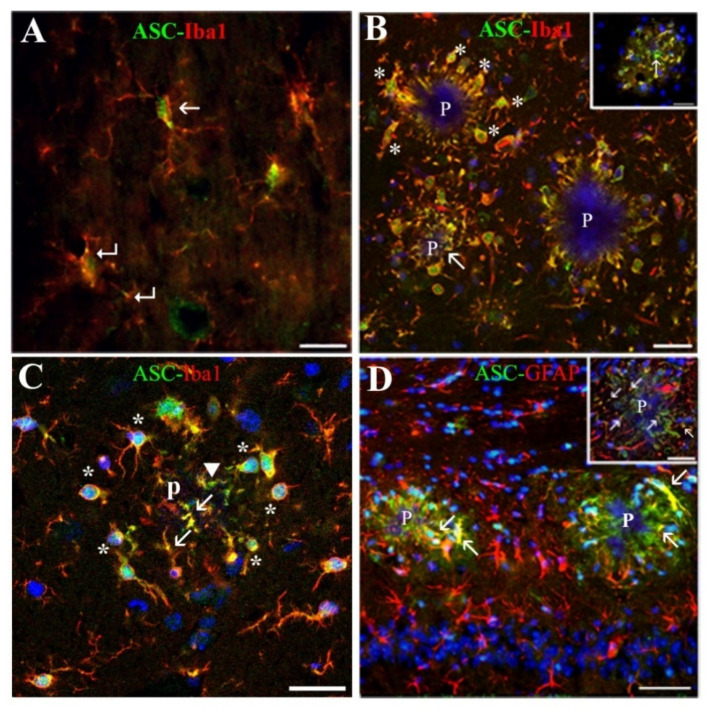
ASC is associated with microglial and astroglial cells soma and processes inside and around plaques. (**B**,**C**) and insets in (**B**,**D**) are confocal images. (**A**) Shows that in WT HP, ASC is mainly located in microglial nuclei (arrow) and in dispersed small spots along their processes (bent arrows), whereas in APP/P HP shown in (**B**,**C**), ASC is present, not only in microglial nuclei (asterisks) but more abundantly in processes penetrating plaques (p) (arrows in (**B**,**C**). Note that some ASC inside the plaques does not co-label with Iba1 (arrow in (**B**) inset, and arrowhead in (**C**)), and that microglial nuclei are white due to co-labeling of ASC and Hoechst. (**D**) Shows that some ASC associated to plaques (p) co-label with astrocytic GFAP (arrows), in soma, processes, and, as shown in (**D**) inset, also in process terminals (arrows) inside the plaque. Scale bars represent 20 µm in (**A**), 50 µm in (**B**) and insets, and 25 µm in (**C**,**D**).

Microglial cells present in WT and in APP/D expressed ASC principally in their nuclei (Figure 2A, arrow), but a small amount was also detected in their processes (Figure 2A, bent arrows). However, as shown in confocal images in APP/P (Figure 2B,C), there was an increase of ASC in microglia associated with Aβ plaques, so that an important amount of protein was detected, not only in nuclei and soma (Figure 2B,C, asterisks) but also in many cell processes directed towards or penetrating into the plaques (Figure 2B,C, arrows). Since there was an increase in the number of microglial cells in hippocampal regions displaying plaques, these regions contained a higher amount of ASC and Iba1 than the corresponding regions in WT (compare Figure 2A,B) and APP/D.

As shown in Figure 2B, we frequently found Aβ plaques stained with Hoechst after nuclear staining of tissue sections. This fact, which probably depended on our methodological procedures, allowed us to visualize plaques at the same time as two other cellular or protein immuno-markers. Most of the ASC present around and inside the plaques co-localized with Iba1 (yellow fluorescence in Figure 2B,C, arrows), even in microglial processes present in the center of the plaque (Figure 2C, arrows). Nevertheless, there was still ASC inside and around the plaque in other extra-microglial locations (Figure 2B inset, arrow; Figure 2C, arrowhead). It was noteworthy to find that some astroglial cells also contained ASC inside their GFAP-positive cell bodies, in their processes penetrating into the plaques (Figure 2D, arrows), and in their process terminals, as we confirmed in confocal z-stack images (Figure 2D inset, arrows).

**Figure 3:**
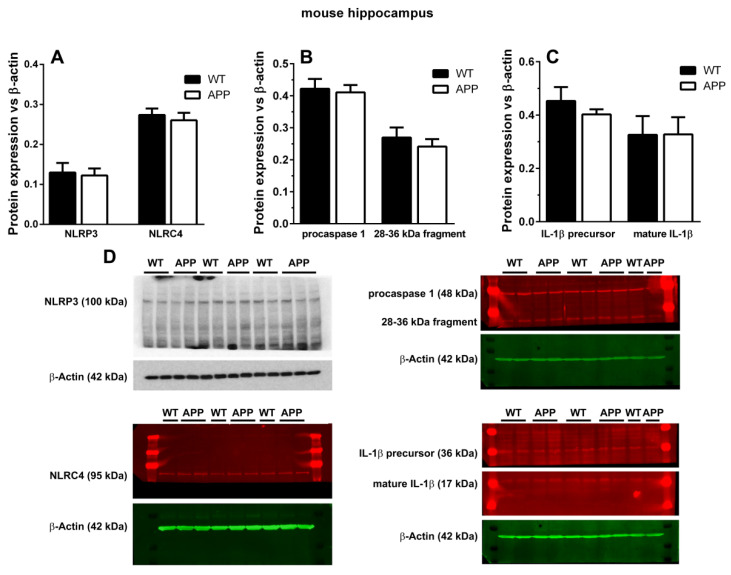
No changes in the expression of neural components of active inflammasomes and of mature IL-1β were detected in HP of APP/P versus WT. Panels (**A**–**C**) show quantitative Western blot analysis of NLRP3 and NLRC4 (**A**), proforms of caspase-1 (**B**), and IL-1β precursor and its active form (**C**). Note that there are no significant changes of these proteins in APP/P versus WT. (**D**) shows a representative Western blot of NLRP3, NLRC4, procaspase-1, Il-1β precursor, mature IL-1β, and their respective β-actin as charge control. In the case of caspase-1, a band of 48 kDa, which corresponds to procaspase-1, and another band below 36 kDa ((**B**,**D**), 28–36 kDa fragment) are observed. Results represent the mean ± SEM of 3 to 5 independent mice in each experimental group.

Since microglial ASC could be associated with IF, we analyzed in these cells the expression of several cytoplasmic PRR of the NLR family: NLRP1, NLRP3, and NLRC4, which have been detected in neural cells. However, antibodies used by us for immunodetection of these receptors did not showspecific staining neither in microglial cells associated with plaques nor in the plaques themselves (data not shown). Since these results were unexpected, we analyzed through Western blot the expression of NLRP3 and NLRC4 in WT and APP/P HP (Figure 3A,D). In addition, we studied through this technique the expression of the proforms and mature forms of caspase-1 and IL-1β (Figure 3B–D), since an increase in the ratio of mature forms versus proforms would be indicative of the presence of active IF. The bands of most of the proteins had the corresponding molecular weight referred to in the literature (Figure 3D). However, although procaspase-1 was readily detectable at ~48 kDa, in the case of caspase-1, which molecular weight is ~18 kDa, we could only visualize a band between 28–36 kDa but, there were no bands visible below 28 kDa. We refer to this protein as the “28–36 kDa fragment”, which probably corresponds to the 33–35 kDa active caspase-1 described by others [32,33]. In any case, and in accordance with the immunohistochemical results, as shown in Figure 3, no changes in the expression of any of these proteins were observed by Western blot in APP/P versus WT HP.

### 2.2. A Subpopulation of Hippocampal Astrocytes Not Related to Aβ Plaques Expressed ASC and Were Associated to Foci of Extracellular ASC Granules in the HP of Aged WT and APP. The Frequency of Mice Generating These Foci Was Almost 3 Times Higher in APP Than in WT

**Figure 4:**
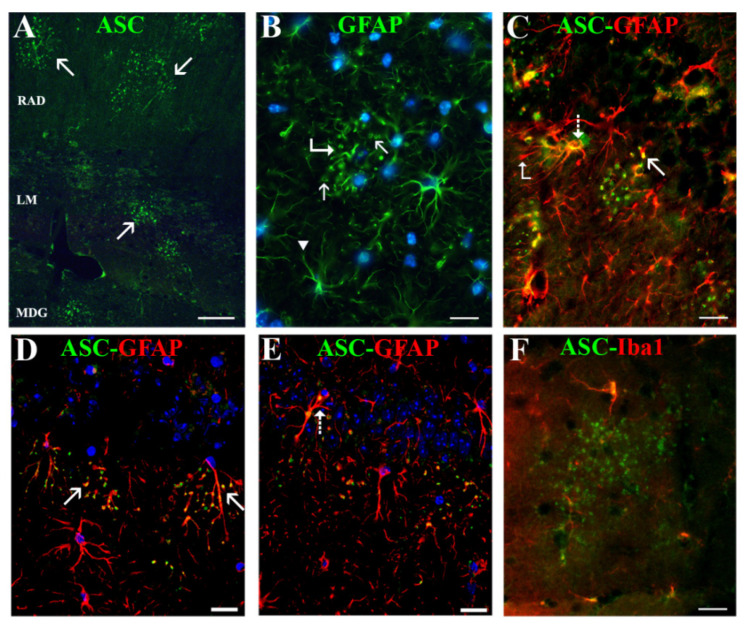
APP frequently show foci of ASC granules dispersed throughout the HP, intimately associated withspecific astrocytes, which also contain ASC in their cytoplasm. (**D**,**E**) are confocal images of a same z-stack. (**A**) shows a panoramic view of three distinct strata in the HP of an APP/D, i.e., strata radiatum (RAD), lacunosum- molecularis (LM), and molecularis of DG (MDG), which present distinct foci of ASC granules (arrows). (**B**,**C**) reveal the appearance of specific GFAP-positive astrocytes (bent arrow in (**B**)) associated with these ASC granules (arrows in (**C**,**D**)), which have swellings along their processes (bent arrow in (**C**)) and terminate in loops (arrows in (**B**)) instead of the more common tapered terminals (arrowhead in (**B**)). The intimate association of these granules with astrocytic processes is seen in (**D**) (arrows) and (**E**), and the presence of ASC next to the nucleus and in primary processes is seen in (**C**) and (**E**) (dashed arrows). (**F**) shows no apparent association between ASC granule clusters and Iba1-positive microglia. Scale bars represent 50 μm in (**A**), and 20 μm in (**B**–**F**).

The density of microglial cells in APP/D HP developing diffuse Aβ deposits was similar to WT mice (Figure 1E). In addition, this cell type, as well as astrocytes, showed a uniform distribution throughout APP/D hippocampal strata, which was similar to WT, suggesting an absence of glial activation in response to diffuse deposits. Instead of showing ASC associated with Aβ plaques, this group of mice frequently developed foci with clusters of apparently extracellular-located ASC granules with a diameter of 1–3 µm. In coronal sections, ASC granules were located in strata RLM (Figure 4A), and less frequently in stratum oriens, but were not present in other cerebral regions visualized in the sections herein analyzed (cortex, thalamus, hypothalamus, caudate-putamen nucleus, amygdale formation). These foci also developed in APP/P mice, where they were not spatially related with Aβ plaques, and, in fact, less visible since most ASC was associated to Aβ plaques in the HP of these mice.

In order to discard that the ASC immunostaining of these granules could be a false positive staining, due to the possible presence of IgM antibody contaminants in the commercial anti-ASC antibody used by us (see discussion), we treated sections, which had been previously incubated in rabbit polyclonal anti-ASC antibody, with secondary goat anti-rabbit IgM antibodies conjugated to fluorescein (1/50, ab98458, Abcam, Cambridge, MA, USA). This procedure gave no positive immunostaining in the granules identified by us (data not shown), revealing that this ASC immunostaining was not due to IgM contaminants which could be present in the anti-ASC antibody.

Interestingly, the foci of ASC granules were associated with a specific type of astrocyte (Figure 4B, bent arrow and Figure 4C), characterized by swellings or rings in its process terminals (Figure 4B arrows, and 4C, bent arrow), distinct from the tapered process terminals of normal astrocytes (Figure 4B, arrowhead). The ASC granules appeared inside these terminal widenings, but also, as determined by confocal microscopy, inside the processes, dispersed along their length (Figure 4D, arrows, and Figure 4E), which thereby acquired a varicose appearance. Uniform, not punctated, ASC staining was also frequently present in the thicker primary processes (Figure 4E, dashed arrow) or next to the nucleus of these astrocytes (Figure 4C, dashed arrow). In addition, each of these astrocytes was surrounded by many ASC granules which were extracellular, since they did not colocalize with GFAP in successive confocal optical sections across them (Figure 4D,E), neither with microglial Iba-1 (Figure 4F). We were unable to demonstrate NOD-like receptors in ASC granules. NLRP1 and NLRP3 were not detected, and NLRC4 showed an immune-positive staining, but we confirmed that in this case, that it was due to an IgM contaminant in the primary antibody that bound unspecifically to the granules (data not shown).

**Figure 5:**
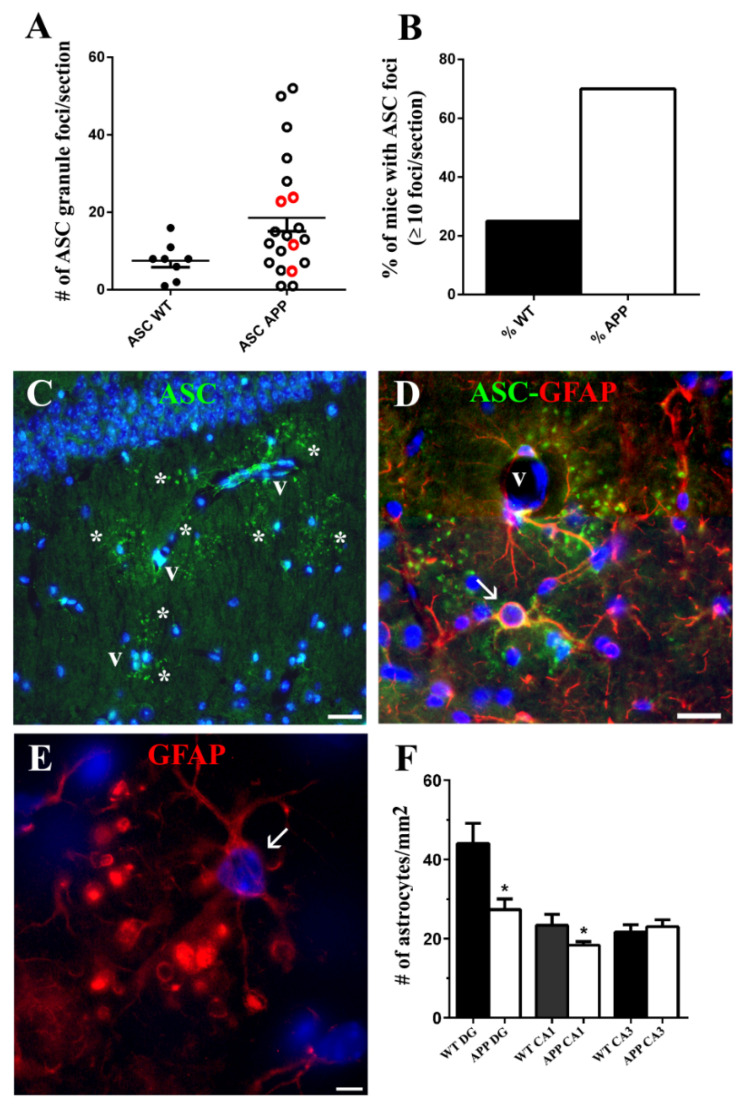
ASC foci development in HP is associated with blood vessels. Alteration of ASC granule-associated astrocytes might account for the decrease in astroglial density in APP radiatum. (**A**) shows a scatter plot of individual values corresponding to the total number of ASC granules foci per section in HP and DG in 8 WT and 20 APP (4 APP/P and 16 APP/D marked with red and black unfilled points, respectively). Note the tendency towards an increase in the number of ASC foci in APP. The graph in (**B**) shows that the percentage of mice developing ≥10 foci per coronal section in APP was around three times higher than in WT. (**C**) reveals that as many as 8 ASC foci (asterisks) are located around a blood vessel (v) segment, and (**D**) shows a perivascular astrocyte (arrow) containing ASC in its soma and process, surrounded by ASC granules. (**E**) shows an ASC-associated astrocyte (arrow) with prominent changesin the process terminal morphology. The graph in (**F**) indicates a significant decrease in the density of astrocytes in the strata radiatum of HP and hilus of DG of 3 APP versus 3 WT. In (**A**,**F**) the symbol # represents the number of ASC granule foci (**A**) and the number of astrocytes (**F**). Student’s test comparison between columns. * *p* ≤ 0,05 vs. WT. Scale bars represent 30 μm in (**C**), 20 µm in (**D**), and 5 µm in (**E**).

We quantified the number of ASC specks foci per hippocampal coronal section in 8 aged WT and in 20 aged APP (4 APP/P and 16 APP/D, in Figure 5A red and black unfilled points, respectively). Although no significant changes were found between WT and APP data, there was a clear tendency towards an increase in the number of ASC foci in the latter group (7/20 values of APP were above the maximal value of WT; two of these APP higher values corresponded to APP/P, whereas the remaining five values were from APP/D). In addition, when we calculated from the pool of animals exhibiting foci of ASC, the percentage of animals developing ≥10 foci per coronal section, the value in APP was around three times the value in WT (Figure 5B), reinforcing the association between the development of ASC foci and the disease. However, when we calculated the percentage of animals developing ASC foci per coronal section in the groups APP/P and APP/D, values were 42.85%, versus 64.7%, respectively, indicating that the generation of plaques was not directly correlated with that of foci of ASC. In fact, the highest number of ASC foci was detected in the APP/D experimental group.

ASC granules and astrocytes associated with them were frequently located next to blood vessels in strata RLM (Figure 5C,D). In coronal sections of the stratum radiatum, it was possible to find microvessel stretches surrounded by 6 to 8 foci of ASC-specks (Figure 5C, asterisks), associated with perivascular astrocytes with the specific morphological characteristics previously described (Figure 5D, arrow).

We also analyzed the incidence of astrocytes associated with ASC granules in the CA1 of those aged APP/D presenting the highest number of ASC foci. Quantification was performed in five randomly selected areas of the stratum radiatum of each hemisphere in coronal sections of 4 APP/D. Among the total astrocytic population, the percentage of these specific astrocytes per section was 21.3 ± 2.57, which was a relatively high proportion. In addition, several of these astrocytes suffered a morphological change in their process terminals, which shifted from tapered to enlarged ring-shaped or spherical terminals with apparent inclusions inside them (Figure 5E). Since these changes and/or the presence of ASC in the cytoplasm of these cells (Figure 4C–E) [23] might be deleterious for them, we quantified the number of astrocytes per mm^2^ in CA1 and CA3 regions of the stratum radiatum and in the DG hilus of 3 WT and 3APP/D hippocampal coronal sections. Results showed that in APP/D hilus and stratum radiatum of CA1 there was a decrease in the astrocyte density versus WT, which was significant when a comparison of columns with a Student´s t-test was carried out (Figure 5F).

**Figure 6:**
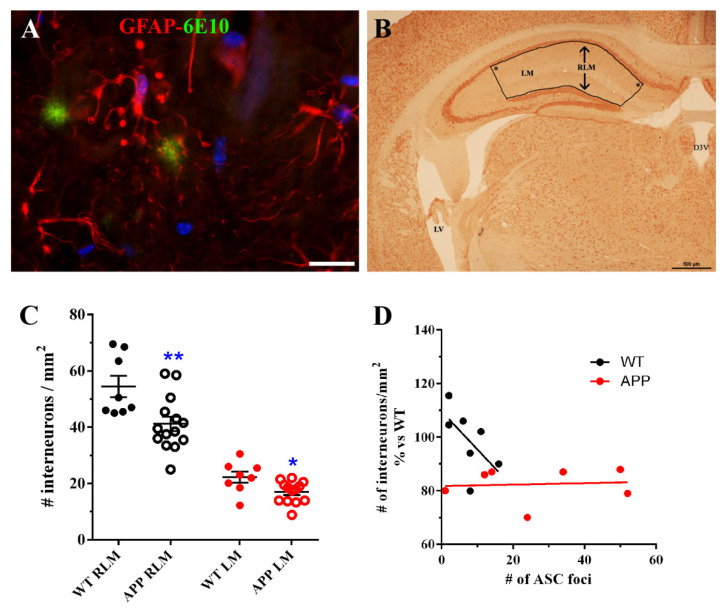
The number of interneurons per mm^2^ localized in the strata RLM of HP is significantly reduced in APP versus WT. This reduction is not correlated with the amount of ASC foci developed in these regions. (**A**) shows a representative specific astrocyte associated to diffuse Aβ deposits, with spherical process terminals in proximity to diffuse Aβ deposits. (**B**) shows a representative coronal section at ~−2mm of Bregma, showing the bounded area used for the quantification of NeuN-positive interneurons in the strata RLM or only in LM in 6 WT and 13 APP. D3V: dorsal third ventricle, LV: lateral ventricle, * right angles in the bounded area (see methods). (**C**) shows this quantification as a scatter plot, where each value represents the mean ± SEM of interneuronal densities from both hippocampal hemispheres of each analyzed coronal section. Note the significant reduction of interneurons in APP both in RLM and LM. (**D**) shows that there is no correlation between the density of interneurons in RLM and the number of foci/section developed in 7 APP with the highest number of ASC foci. By contrast, in 7 WT there is an inverse correlation between both parameters, though the number of foci is smaller. In (**C**,**D**) the symbol # represents the number of interneurons and ASC foci. Student´s test. * *p* ≤ 0.05 and ** *p*≤ 0.01 vs. WT. Scale bars represent 20 μm in (**A**) and 500 µm in (**B**).

From our results, we conclude that the development and distribution of ASC foci did not seem to be related to Aβ plaques. Though some of our observations indicated that the presence of diffuse Aβ deposits could be spatially related with the distribution of the specific astrocytes herein described (Figure 6A), this hypothesis should be further analyzed.

Finally, we considered the possibility that ASC foci in strata RLM of HP in APP mice could be related to an alteration of interneurons distributed in these strata. Therefore, we performed a quantification of NeuN-positive interneurons in these strata (Figure 6B) in 6 WT and in 13 APP with ≥0 ASC foci per section. Results show that there was a significant reduction (*p* < 0.01; Student´s test) in the number of interneurons per mm^2^ in the strata RLM of APP versus WT (Figure 6C). When only the values of interneurons localized in the stratum lacunosum-molecularis (LM) were compared, a significant reduction (*p* < 0.05; Student´s test) was also observed (Figure 6C), so that the depletion in the strata RLM was in part due to the death of interneurons in the LM of APP hippocampi. However, when we studied the possible correlation between the number of interneurons and the number of foci in APP/D, the regression line did not indicate any correlation between both sets of data (Figure 6D). Only in the WT group, where the number of foci was less than 20, there was an inverse correlation between the ASC foci and the number of interneurons (Figure 6D).

## 3. Discussion

Among the HP of the 27 old-aged APP herein analyzed, we have found 7 HP characterized by the presence of both compact Aβ plaques and diffuse Aβ deposits (APP/P), versus 20 HP which developed mainly diffuse Aβ deposits and scarce, if any, plaques (APP/D). The 8 old-aged WT did not exhibit any type of Aβ deposits. Both types of amyloid deposits have been previously described in Tg2576 transgenic mice [29,31], and there does not appear to be a temporal or spatial relationship between diffuse and compact forms, since the former seem not to be a precursor of the latter [31,34]. We used the 6E10 antibody, recognizing specifically the amino acid residues 1–16 of human Aβ and, thereby, the isoforms Aβ_1-40_, Aβ_1-42_,and the precursor form APP, to detect both Aβ plaques and diffuse amyloid deposits in HP. The latter showed a characteristic fibrillar appearance in immersion-fixed brains, as has also been described by some authors [34,35], but not by others, who observed a more amorphous structure [36]. Since these deposits consist only of Aβ [34,36], these differences may be related to the fixation procedure. In fact, in our hands, the morphological features of diffuse deposits were not accurately visualized in perfusion-fixed APP brains but required immersion fixation of the tissue.

The high number of APP with diffuse deposits and relatively few or without plaques (<10 plaques/mm^2^) (APP/D) could be very likely due to a low expression level of the mutated APP transgene in this group of mice versus the APP/P. In fact, the mice used in the present study originated after many crosses between animals lacking the APP transgene and those expressing it, so that the dose of the transgene in APP was probably diluted since it is a function of the number of crosses between animals.

Similar to previous reports [34,36,37], in our study both microglia and astroglia displayed a different function, depending on the type of amyloid deposit to which they were associated. The microglia of APP/D HP did not respond to diffuse amyloid deposits with activation, as also referred by other authors [37], so that APP/D HP showed similar microglial density and microglial and astroglial morphological features to the WT HP. Therefore, we questioned if these old-aged APP/D had indeed any other alteration in the HP. In this study, we show a significant reduction in the density of astrocytes and interneurons in APP/D HP when compared to those in WT HP. In addition, the percentage of animals developing ASC foci in the HP was higher in APP/D than in their APP/P counterparts (64.7% versus 42.85%), and the highest number of ASC foci/section corresponded to APP/D, but not to APP/P.Thus, although we did not analyze their behavior, old-aged APP/D exhibit hippocampal alterations that may influence the cognitive performance of this group of mice. In fact, another APP murine model developing only diffuse amyloid deposits acquires specific spatial learning deficits with age [38].

Microglial cells of APP/P HP associated with Aβ plaques, showed a polarized orientation of their processes, which could penetrate into or partially encapsulate the plaque, exhibiting very probably a phagocytic or plaque compaction activity, respectively, as has been referred in other mouse AD models [39]. In this location, the ASC protein was expressed by all microglial cells in their nuclei, and by these cells and some astrocytes in their soma, prolongations entering the plaques and process terminals, as evaluated in confocal z-series optical sections. Therefore, a relatively high amount of ASC was seen inside the plaques. Our results are in line with other studies referring that microglia, which expresses ASC constitutively, responds to certain danger signals as Aβ with cytoplasmic aggregates of activated ASC [15,40]. In the case of astrocytes, the induction of ASC in these cells has also been recently reported in mice primary cultures primed with LPS and incubated with Aβ [18], but, to our knowledge, has not been described before in in vivo AD models. Interestingly, an elevated expression of ASC has been recently reported in AD human HP using Western blot techniques [41].

Regarding the function of this increase in ASC, some authors have referred that the amount of this molecule in microglial cells is inversely correlated with their Aβ phagocytic activity and production of proteolytic enzymes for Aβ clearance [1,18]. This could be the case in the present old-aged APP/P HP. The anti-phagocytic effect of ASC is associated withthe activation of IF, where the molecule recruits procaspase-1, generating active caspase-1 [4,11,13]. However, when we analyzed the possible presence of cytosolic NOD-like receptors of danger signals in microglial cells surrounding plaques, we could not detect any specific staining for NLRP1, NLRP3, or NLRC4 in these cells, nor in astroglial cells associated with plaques. Since these results were unexpected, we complemented them with Western blot analyses, but we could not find any change in the expression levels of NLRP3 and NLRC4 between APP/P and WT, though these old-aged transgenic mice were characterized by an important increase in ASC when the same Western blot technique was applied [10]. There were no differences in the relative optical densities of caspase-1 and IL-1β proforms and in mature IL-1β between both experimental groups. Despite performing several Western blots, we did not visualize the ~18 kDa cleaved caspase-1, but, instead, we found a 28–36 kDa fragment that could match with the 33–35 kDa caspase-1 subunit observed in human monocytes [32] and mouse macrophages and neutrophils [33]. In fact, it has been demonstrated that this subunit corresponds to an active caspase-1 species [33]. We also found no differences in the levels of this protein between WT and APP mice.

These results seem to be in contradiction with previous in vivo studies, where the authors immunohistochemically detect NLRP3 in microglia surrounding Aβ plaques in the HP of 16 month-old APP_swe_PSEN1_dE9_ mice, using the same anti-NLRP3 antibody as us [1]. Since the authors infer from this result and other experiments the presence of NLRP3 IF in microglial cells associated with plaques, we do not include in the manuscript a confocal image exhibiting the co-localization of ASC and NLRP3 in these cells. On the other hand, the differences in the immunodetection of NLRP3 in their results versus ours may be due, not only to different tissue processing and immunohistochemical procedures but, more importantly, to the use of a different animal model of AD, since our results were obtained from 22 month-old single-mutated APP_swe_ mice, while Heneka et al., 2013, analyzed 16 month-old double-mutated APP_swe_PSEN1_dE9_ mice. We do not know which is the function of ASC present in microglia and astrocytes associated with plaques in the old-aged APP herein studied. However, we can predict that, if this protein is released into the extracellular space, for example through microglial death, it could promote new Aβ deposition and Aβ cross-seeding, as has been shownin an important recent contribution in double mutant APPswePSEN1dE9 [21].We did not analyze if glial cells containing ASC in Aβ plaques died, but, since ASC is implicated both in apoptotic and in pyroptotic cell death [13,25,26], further studies, as TUNEL analysis or Gasdermin D immunodetection, would be of interest.

We discovered a new, not previously reported, ASC distribution not related to Aβ plaques in the cytoplasm of a specific type of hippocampal astrocyte associated with extracellular granules containing also this protein. The granules, which resembled ASC specks described by other authors [23,42], were visualized with the anti-ASC antibodies from Santa Cruz Biotechnology Inc., and with those from Acris Antibodies GmbH, but not with the specific antibody from Adipogen Corp. Since different ASC isoforms have been discovered [43], the possibility exists that the two former antibodies reacted with an ASC isoform which was not recognized by the latter antibody. ASC-positive granules were grouped in discrete clusters or foci dispersed throughout the strata RLM and were absent in the extra-hippocampal regions present in the coronal sections herein analyzed (cortex, thalamus, hypothalamus). Serial optical sections obtained through confocal microscopy confirmed that the ASC granules were extracellular and surrounded specific astrocytes, or were contained inside their secondary processes or process terminals. This latter disposition gave the astrocytes a special morphology characterized by process varicosities and ring-shaped or spherical terminals. With the exception of some astrocytes surrounding amyloid plaques, from all the hippocampal astroglial population herein analyzed only these specific astrocytes associated withASC granules expressed ASC in their cytoplasm

The foci of ASC granules were related to the advanced age of mice, since they developed also in 20-month-old WT, but were not present in 10-month-old APP and WT nor in 3-month-old WT (data not shown). However, the incidence of animals developing ≥10 ASC granule foci per coronal section was nearly three times higher in old-aged APP than in old-aged WT. In spite of this correlation, ASC granule foci were not spatially related to Aβ plaques and were also present in WT, which had no amyloid deposits. In fact, the incidence of foci was especially remarkable in the APP/D group with few or no plaques, which included HP exhibiting the highest number of ASC foci. Therefore, we cannot consider that these ASC foci might be the origin of Aβ plaques.

Several studies have revealed that brain aging in mice leads to a progressive appearance and expansion of extracellular granular structures containing high levels of polysaccharides, which have a complex chemical and ultrastructural composition [44]. Their arrangement in clusters, hippocampal distribution, and association to astrocytes are very similar to those of ASC granules described in the present study. Since the authors demonstrate that IgM antibodies, which are contaminants in numerous commercial preparations of IgG antibodies, tend to bind to these granules showing false immunohistochemical positive staining, we analyzed if the ASC immunostaining detected by us, in granules, was true and not due to the presence of these contaminants. From our results, we conclude that the granular structures described by others, and observed by us in our study, in fact contain ASC. It is possible that the complex chemical nature of these granules might be due to the non-specific but stable co-aggregating properties of diverse cytosolic proteins on ASC specks [45]. By contrast, the NLRC4 immunopositive staining that we also detected in ASC granules was unspecific, since it was due to an IgM contaminant in the primary antibody that bound to the granules. We also did not detect NLRP1 or NLRP3.

The pathogenesis of ASC granules in the hippocampus of old-aged mice and the exacerbation of these structures in APP mice is for us unknown, but they might be a result of inflammatory events and pyroptotic cell death in this area of the central nervous system. There was a remarkable association of foci of ASC granules with some blood vessels of the HP. It has been demonstrated that the paravascular system for cerebrospinal and interstitial exchange of waste products slows down with age and possibly in AD [46,47]. Since this pathway, which is probably the physiological way of clearance of ASC granules from the HP, is very likely impaired in the old-aged APP mice of this study, ASC granules might accumulate extracellularly, next to perivascular astrocytes, as we in fact observed. In this location, they might be sensed as a danger signal by these astrocytes, which might capture them in order to remove them from the extracellular space, similarly to the described behavior of macrophages [23,48]. This clearance process might damage or even kill these ASC-associated astrocytes through pyroptosis, and, in turn, these damaged cells might release ASC specks to the extracellular parenchyma, thereby propagating this pathology in a prion-like manner. In fact, in some strata radiatum of APP/D HP herein analyzed these specific astrocytes represented as much as 20% of all astrocytes, strongly suggesting an outcome of chronic inflammation. Especially in those cases where these astrocytes were abundant, some of them showed features compatible with damage, as aberrant enlargement of the ring-shaped process terminals containing inclusions. It is possible that these features do not reflect death but rather an atrophy, which is, together with reactivity, the reported astrocyte damage associated with AD [49]. However, since we have found a significant decrease versus WT of astrocyte density in the stratum radiatum of the HP in APP/D, the possibility exists that ASC-associated astrocytes ultimately die. The decrease inastrocytes with AD progression over age has also been detected by others in the HP and prefrontal cortex of experimental AD transgenic mice [50]. Age in itself does not seem to vary the number of astrocytes in neocortices of the human brain or in the HP of 21-month-old rats, though the volume of astroglial domains increases about 2 times [50].

In an effort to know which implications our findings could have on the neuronal population, we quantified hippocampal interneurons present in strata RLM of old-aged APP/D and WT HP. Surprisingly, we found that the number of these neurons was significantly reduced in APP versus WT. We found also a reduction when only interneurons in which soma was located in the LM were quantified. To our knowledge, our data on the reduction of interneurons in HP inmurine models of AD, have not been reported previously. Nevertheless, our results should be considered with caution since we were unable to use a stereological approach in the neuronal quantification method (see Materials and Methods) due to a small amount of sample at the time we designed these experiments. Thus, the present results must be further verified in a more accurate future study. It is known that neurons of the human entorhinal cortex that send projections to the CA1 HP are the first degenerating cells in AD [51,52]. The LM is an area of integration and serves as a relay between the entorhinal cortex and the CA1 HP. Since interneurons in this layer are likely to modulate the strength and temporal structure of entorhinal-CA1 hippocampal dynamics, influencing distinct aspects of spatial and episodic memory [53], the partial lack of these cells must influence the HP circuitry and the behavior of old-age APP. On the other hand, we reasoned that the reduction ininterneurons in strata RLM could be related to the presence of deleterious ASC granules associated to astrocytes in the same strata of HP. However, our results indicated that there was no correlation between number of ASC foci and the density of interneurons in these APPs.

In conclusion, our results in the HP of old-aged APP mice showed a relatively large amount of ASC in Aβ plaques, which was of microglial and astrocytic origin and apparently not associated with PRR commonly described in glial cells. In addition, we revealed that the development of foci of ASC granules dispersed throughout the HP, which incidence was higher in old-aged APP than in their WT counterparts, and which were not spatially related with Aβ plaques but to a specific type of astrocyte. Our results suggest the implication of these astrocytes in ASC clearance, and in the propagation of the inflammation mediated by this protein

## 4. Material and Methods

### 4.1. Ethics Statement

The Ethics Committee of the Hospital Ramón y Cajal, Madrid (animal facilities ES280790002001) approved all the protocols related to the use of laboratory animals. All procedures associated with animal experiments were in accordance with Spanish legislation (RD 53/2013) and the European Union Council Directive (2010/63/EU).

### 4.2. Transgenic Mice

The Tg2576 line over-expressing human APP695 was used as a model of AD in this study. This line, generously donated by Dr. Carro [54], contained the human mutated APPswe that included the double mutation M671L and K670N under the control of the prion protein promoter [29]. Mice developed age-dependent AD-type neuropathology [29]. An intercross between APP Tg2576 and wild-type (WT) C57BL6 was performed in order to obtain WT littermate controls. Transgenic animals were generated by breeding the mice according to the following diagram (Equation (1)):

♂APPswe × ♀ WT = APPswe (50/57.6) and WT (50/42.4)(1)

(the numbers in brackets show the expected/found genotype frequencies of the offspring expressed in percentage). For the present study, 13 WT and 38 transgenic mice (APP) of 20 to 22 months of age were used.

### 4.3. Genotype by PCR

The genotype of each animal was confirmed as described by Carro et al. [54]. Genomic DNA was extracted from the mouse tail using the High Pure PCR template preparation kit (Roche, Barcelona, Spain) according to the manufacturer’s instructions. Briefly, 150 ng of DNA was amplified by polymerase chain reaction (PCR) using the specific primers described in [54] to detect mutant human APP sequence.

### 4.4. Antibodies and Immunochemicals

The primary antibodies used were: three different rabbit polyclonal anti-ASC antibodies: (1:50, sc-22514-R, Santa Cruz Biotechnology Inc., Dallas, TX, USA), (1:50, AP06792PU-N, Acris Antibodies GmbH, Herford, Germany), (1:50, AG-25B-0006, Adipogen Corp., San Diego, CA, USA); mouse monoclonal anti-Aβ antibody (6E10, 1:100, SIG-39320, Covance, Emeryville, CA, USA); two anti-Iba1 antibodies: rabbit polyclonal (1:100; Wako Chemicals USA, Inc., Los Angeles, CA, USA), mouse monoclonal (1:200, MABN92, Millipore Corp., Massachusetts, MA, USA); mouse monoclonal anti-NLRP3 antibody (1:200, AG-20B-0014, Cryo-2, Adipogen San Diego, CA, USA); two different anti-NLRC4 antibodies: goat polyclonal (1:25, sc-49395, Santa Cruz Biotechnology Inc., Dallas, TX, USA), rabbit polyclonal (1:50, AVARP00019-P050, Aviva Systems Biology, San Diego, CA, USA) two anti-glial fibrillary acidic protein (GFAP) antibodies: rabbit polyclonal (1:200, 20334, DakoCytomation, Glostrup, Denmark), mouse monoclonal (1:400,Mab360, Millipore Corp., Massachusetts, MA, USA); mouse anti-neuronal nuclei (NeuN) (1:100, Mab377, Chemicon International Inc., Temecula, CA, USA).

The secondary antibodies used were: Alexa Fluor-568 goat anti-mouse IgG, Alexa Fluor-488 goat anti-rabbit IgG, and Alexa Fluor-568 donkey anti-goat IgG (1:400, Molecular Probes, Eugene, OR, USA); fluorescein-conjugated goat anti-mouse IgG (1:25, Jackson Immuno Research Laboratories Inc., West Grove, PA, USA); rhodamine-conjugated goat anti-rabbit IgG (1:100, Chemicon International Inc., Temecula, CA, USA); fluorescein-conjugated goat anti-rabbit IgM mu chain (1:50, ab98458, Abcam, Cambridge, MA, USA), fluorescein-conjugated rabbit anti-goat IgM (1:200, ab112861, Abcam, Cambridge, MA, USA).

### 4.5. Tissue Processing and Immunohistochemistry

The brains of 8 WT and 27 transgenic APP mice were fixed through intracardial perfusion under deep anesthesia with 10 mL of heparinized isotonic saline, followed by 40 mL of 4% paraformaldehyde (PFA). Brains were post-fixed in the same solution for 24 h at 4 °C, cryoprotected and frozen, before sectioning into 20 µm-thick coronal sections in a cryostat. In addition, we processed the brains of 5 WT and 11 APP through fixation immersion with 4% PFA, since with this procedure we noticed that the visualization of diffuse Aβ deposits was more accurate. Fixed brains were subsequently cryoprotected, frozen, and sectioned as above. For quantitative studies of immunostained cells, structures, or molecules in the dorsal HP, only coronal sections of the perfusion-fixed brains were analyzed, located at similar antero-posterior levels, i.e., −1.7 to −2 mm from Bregma [55].

Tissue sections were mounted on coated FLEX IHC microscope slides (DakoCytomation, Glostrup, Denmark), treated with sodium acetate 10 mM, pH 6.0, at 95°C for 4 min, and blocked with 5% normal goat serum (NGS) (or 5% normal donkey serum (NDS) in the case of goat anti-NLRC4 antibody) in 0.1% Triton-X 100 in phosphate buffer saline (PBS), pH 7.4, for 30 min. Primary antibodies diluted in 0.5% NGS or NDS in 0.01% Triton X-100 in PBS were applied for 24 h at 4°C, and were visualized using appropriate fluorescent secondary anti-rabbit, anti-mouse or anti-goat antibodies diluted in 0.5% NGS or NDS in 0.01% Triton X-100 in PBS, during 1 h at room temperature. In order to stain cell nuclei, tissue sections were incubated in 30μM bisbenzimide (Hoechst 33342; Sigma-Aldrich, Saint Louis, MO, USA) in PBS for 5 min, and were thereafter washed in PBS and coverslipped in a medium containing p-phenylenediamine. For double immunolabeling, both primary antibodies directed to two different species were applied at the same time to the sections after the pretreatment steps, followed by incubation of the sections with the appropriate secondary antibodies. Care was taken when selecting secondary antibodies for double immunostainings in order to avoid cross reactions. Since our secondary antibodies were mostly made in goats, goat polyclonal anti-NLRC4 antibody was not used for double immunostainings. In the case of Aβ immunodetection in plaques, sections were pretreated with 70% formic acid for 20 min at RT, followed by the blocking step. In double immunolabeling experiments, Aβ detection was performed after the immunohistochemical procedure for visualization of the other antigen was completed.

An immunoperoxidase technique was used for NeuN detection. The treatment steps previous to primary antibody incubation were as above, but a step for endogenous peroxidase quenching was added before the blocking step (incubation in 1% hydrogen peroxide in PBS for30 min). For the secondary antibody incubation, a biotinylated goat anti-mouse IgG (BA-9200, 1:300, Vector Labs, Burlingame, CA, USA) was used during 1 h at room temperature, followed by a Peroxidase-Streptavidin Conjugate (1:200, 43-4323, Life Technologies, Carlsbad, CA, USA) treatment during a similar period. Finally, the reaction product was detected with diaminobenzidine (DAB) + substrate–chromogen system (DakoCytomation, Glostrup, Denmark). In these sections, nuclei were also visualized with bisbenzimide, as described above.

Immunohistochemical staining was analyzed with a standard fluorescence microscope, but for several analyses, a Nikon ECLIPSE Ti-e C1 confocal microscope (Nikon Instruments Inc., Tokyo, Japan) was used and z-stack series were performed.

### 4.6. Quantitative Analysis

For quantification of hippocampal Aβ 6E10-positive plaques, microglial Iba-1-positive cells, and astroglial GFAP-positive cells, a panoramic view of each hemisphere was obtained from one representative coronal section for each immunohistochemical technique and for each brain. In these panoramic views, obtained by using the stitching tool coupled to the Nikon ECLIPSE Ti-e microscope and the 10x objective, the transversal surface of the dorsal hippocampus and dorsal cortex of each cerebral hemisphere could be visualized. We quantified: (a) the number of 6E10-positive Aβ plaques/mm^2^ given as the mean ± SEM obtained from the data of both hippocampal hemispheres in 5 APP mice; (b) the number of Iba-1-positive microglial cells/mm^2^ in 2 different hippocampal areas of each cerebral hemisphere, one of them localized in the polymorphic layer of the dentate gyrus (DG) (hilus), and the other in the strata radiatum and lacunosum-molecularis of the HP, and the stratum molecularis of the DG (RLM). The data in the graph represent the mean obtained from the data of both hippocampal hemispheres in 3 WT, 4 APP with Aβ plaques (APP/P), and 3 APP with diffuse amyloid deposits (APP/D) (Figure 1E); (c) the total number of foci of ASC granules per coronal section of HP and DG, given as the sum of ASC foci of both hemispheres in 8 WT and 20 APP (4 APP/P and 16 APP/D). Animals with no ASC foci were not included (Figure 5A); (d) the number of astrocytes/mm^2^ in 3 hippocampal regions: hilus of DG, radiatum of CA1 and radiatum of CA3 in 3 WT and 3 APP/D (Figure 5F). The number of NeuN-positive interneurons/mm^2^ present in strata RLM or only in LM were quantified in the bounded area (Figure 6B) of hippocampal coronal sections at ~−2mm from Bregma (Figure 6B), in 6 WT and 13 APP/D with ≥10 ASC foci per section. Care was taken when delineating the bounded area, which always had right angles in the locations marked by an asterisk in Figure 6B, in order to assure similar surfaces between different sections where interneurons were quantified. Each data entered in the graph (Figure 6C) represent the mean ± SEM of interneuronal densities from both hippocampal hemispheres of each analyzed coronal section. For the correlation studies between the interneuron density in RLM and the total number of foci of ASC granules per section, 6 WT and 7 APP were selected, including in both groups those animals with the highest number of ASC foci (Figure 6D).

### 4.7. Brain Regions and Tissue Preparation for Biochemical Analysis

The HP of 5 WT and 5 APP/P were used for Western blot procedures. In order to select for this study, only those APP that developed a significant number of plaques in their HP, brains were sagitally sectioned through their midline, so that the right part of the HP was dissected and rapidly frozen for Western blot and the left part of the brain was fixed by immersion in 4% PFA for histological analysis of plaques. Those brains with few or without plaques were discarded.

After decapitation, the brain was extracted and HP was free-hand dissected, then the samples were frozen on dry ice. For protein extraction, dried tissue samples were weighed and placed in six volumes (W/V) of PBS with protease inhibitor cocktail 1× (Calbiochem) and 20 mM N-ethylmaleimide to inactivate deubiquitinating enzymes and subjected to two 30-srounds of sonication. The lysates were immediately boiled for 5 minand centrifuged at 12.000× *g* at 4 °C for 30 min. The supernatant-PBS-inhibitors was defined as the soluble fraction and was used for protein analysis by Western blot. To obtain the total (soluble plus insoluble) fractions, the initial homogenate (200 μL) was extracted with 5 M guanidine in 6 mM Tris–HCl, pH 8.0 by rotating the sample at room temperature overnight. The sample obtained after guanidine extraction represented the total fraction. Soluble and total fractions were used for protein analysis by Western blot.

### 4.8. Western Blot

Samples (20–40 μg) were added to sodium dodecyl sulfate (SDS) sample loading buffer 2× (10% glycerol, 2% SDS, 0.1% bromophenol blue, 50 mM Tris, pH 6.8 and 5% β-mercaptoethanol), electrophoresed in 10–15% SDS-polyacrilamide gels and then electro blotted to 0.45 μm nitrocellulose membranes. For immunolabeling, the blots were blocked with TTBS solution (20 mM Tris-HCl, pH 7.6, 137 mM NaCl plus 0.1% Tween 20, and 5% dry skimmed milk) for 2 h at room temperature. After blocking non-specific binding, the membranes were incubated overnight with specific antibodies in blocking solution at 4 °C. Later, the blots were washed twice with blocking solution for 10 min followed by another two washes with TTBS for 5 min each. To detect the infrared fluorescence of the membranes the ODYSSEY Infrared Imaging System (LI-COR Biosciences) was used. Besides, some membranes were developed by chemiluminescence detection using a commercial kit (Bio-Rad) and images were captured with ChemiDoc Imaging System (Bio-Rad). The density of stained bands was scanned and quantified with the Image QuantTL 1-D software package (GE Healthcare Life Sciences, Uppsala, Sweden) and the data were normalized with respect to β-actin levels.

The primary antibodies used in this study were: goat polyclonal anti-IL-1β (1:250, AF-401-SP, Bio-Techne R&D Systems, SL, Barcelona, Spain); rabbit polyclonal anti-Caspase-1 (1:300, 3019-100 BioVision, Milpitas, CA, USA); mouse monoclonal anti-NLRP3 (1:400, AG-20B-0014-C100, AdipoGen Life Science, San Diego, CA, USA). The secondary antibodies were: IRDye 680RD donkey anti-goat 925-68074 (1:10,000); IRDye 680RD goat anti-rabbit 925-68071 (1:10,000); IRDye 800RD goat anti-mouse 925-32210 (1:10,000) (the three from Li-Cor, Lincoln, NE, USA) and anti-mouse IgG-peroxidase (1:10,000) (Sigma-Aldrich, San Louis, MO, USA).

To correct and quantify the protein load, mouse monoclonal anti-β-actin antibody (diluted 1:10,000) was used from Sigma. The data of the proteins of interest analyzed in the study were normalized with respect to β-actin levels.

### 4.9. Statistical Analysis

Results were expressed as the mean ± SEM of (n) independent animals. Statistical analysis was performed with the GraphPad Prism software (La Jolla, CA, USA). A one-way ANOVA followed by the Newman–Keuls multiple comparisons test were performed. Differences were considered significant when *p* ≤ 0.05.

## Data Availability

Not applicable.

## Abbreviations

Aβ	amyloid beta
AD	Alzheimer´s disease
APP	transgenic mice containing the human mutated APPswe
APP/D	APP mice with diffuse Aβ deposits and ≤10 Aβ plaques/mm^2^
APP/P	APP mice with ≥ 10 Aβ plaques/mm^2^
ASC	apoptosis-associated speck-like protein containing a caspase recruitment domain
CARD	caspase recruitment domain
DG	dentate gyrus
GFAP	glial fibrillary acidic protein
HP	hippocampus
Iba1	ionized calcium-binding adapter protein 1
IF	inflammasome
IL	interleukin
LM	stratum lacunosum-molecularis of hippocampus
NeuN	neuronal nuclei
NLR	NOD-like receptors
NLRP1	NLR family PYD-containing 1
NLRP3	NLR family PYD-containing 3
NLRC4	NLR family CARD-containing 4
NOD	nucleotide binding and oligomerization domain
PRR	pattern recognition receptors
PYD	pyrin domain
RLM	strata radiatum, lacunosum-molecularis of hippocampus and stratum molecularis of dentate gyrus
ROS	radical oxygen species
TGF-β	transforming growth factor-β
TNF-α	tumor necrosis factor-α
WT	wild type

## References

[1] Heneka M.T., Kummer M.P., Stutz A., Delekate A., Schwartz S., Vieira-Saecker A., Griep A., Axt D., Remus A., Tzeng T.-C. (2013). NLRP3 is activated in Alzheimer’s disease and contributes to pathology in APP/PS1 mice. Nature.

[2] Heneka M.T., Carson M.J., El Khoury J., Landreth G.E., Brosseron F., Feinstein D.L., Jacobs A.H., Wyss-Coray T., Vitorica J., Ransohoff R.M. (2015). Neuroinflammation in Alzheimer’s disease. Lancet Neurol..

[3] Tang Y., Le W. (2016). Differential Roles of M1 and M2 Microglia in Neurodegenerative Diseases. Mol. Neurobiol..

[4] Heneka M.T., McManus R., Latz E. (2018). Inflammasome signalling in brain function and neurodegenerative disease. Nat. Rev. Neurosci..

[5] Ennerfelt H.E., Lukens J.R. (2020). The role of innate immunity in Alzheimer’s disease. Immunol. Rev..

[6] Li C., Zhao R., Gao K., Wei Z., Yin M.Y., Lau L.T., Chui D., Yu A.C.H. (2011). Astrocytes: Implications for neuroinflammatory pathogenesis of Alzheimer’s disease. Curr. Alzheimer Res..

[7] Fuller S., Münch G., Steele M. (2009). Activated astrocytes: A therapeutic target in Alzheimer’s disease?. Expert Rev. Neurother..

[8] Sokolowski J.D., Mandell J.W. (2011). Phagocytic Clearance in Neurodegeneration. Am. J. Pathol..

[9] Daria A., Colombo A., Llovera G., Hampel H., Willem M., Liesz A., Haass C., Tahirovic S. (2017). Young microglia restore amyloid plaque clearance of aged microglia. EMBO J..

[10] Gonzalo-Gobernado R., Perucho J., Vallejo-Muñoz M., Casarejos M.J., Reimers D., Jiménez-Escrig A., Gómez A., De Asanza G.M.U., Bazán E. (2020). Liver Growth Factor “LGF” as a Therapeutic Agent for Alzheimer’s Disease. Int. J. Mol. Sci..

[11] Walsh J.G., Muruve D.A., Power C. (2014). Inflammasomes in the CNS. Nat. Rev. Neurosci..

[12] Voet S., Srinivasan S., Lamkanfi M., Van Loo G. (2019). Inflammasomes in neuroinflammatory and neurodegenerative diseases. EMBO Mol. Med..

[13] Platnich J.M., Muruve D.A. (2019). NOD-like receptors and inflammasomes: A review of their canonical and non-canonical signaling pathways. Arch. Biochem. Biophys..

[14] Tzeng T.-C., Hasegawa Y., Iguchi R., Cheung A., Caffrey D.R., Thatcher E.J., Mao W., Germain G., Tamburro N.D., Okabe S. (2018). Inflammasome-derived cytokine IL18 suppresses amyloid-induced seizures in Alzheimer-prone mice. Proc. Natl. Acad. Sci. USA.

[15] Halle A., Hornung V., Petzold G.C., Stewart C.R., Monks B.G., Reinheckel T., Fitzgerald K., Latz E., Moore K., Golenbock D.T. (2008). The NALP3 inflammasome is involved in the innate immune response to amyloid-β. Nat. Immunol..

[16] Gustin A., Kirchmeyer M., Koncina E., Felten P., Losciuto S., Heurtaux T., Tardivel A., Heuschling P., Dostert C. (2015). NLRP3 Inflammasome Is Expressed and Functional in Mouse Brain Microglia but Not in Astrocytes. PLoS ONE.

[17] Liu L., Chan C. (2014). IPAF inflammasome is involved in interleukin-1β production from astrocytes, induced by palmitate; implications for Alzheimer’s Disease. Neurobiol. Aging.

[18] Couturier J., Stancu I.-C., Schakman O., Pierrot N., Huaux F., Kienlen-Campard P., Dewachter I., Octave J.-N. (2016). Activation of phagocytic activity in astrocytes by reduced expression of the inflammasome component ASC and its implication in a mouse model of Alzheimer disease. J. Neuroinflamm..

[19] Bryan N.B., Dorfleutner A., Rojanasakul Y., Stehlik C. (2009). Activation of Inflammasomes Requires Intracellular Redistribution of the Apoptotic Speck-Like Protein Containing a Caspase Recruitment Domain. J. Immunol..

[20] Dick M., Sborgi L., Rühl S., Hiller S., Broz P. (2016). ASC filament formation serves as a signal amplification mechanism for inflammasomes. Nat. Commun..

[21] Venegas C., Kumar S., Franklin B.S., Dierkes T., Brinkschulte R., Tejera D., Vieira-Saecker A., Schwartz S., Santarelli F., Kummer M.P. (2017). Microglia-derived ASC specks cross-seed amyloid-β in Alzheimer’s disease. Nature.

[22] Indramohan M., Stehlik C., Dorfleutner A. (2018). COPs and POPs Patrol Inflammasome Activation. J. Mol. Biol..

[23] Franklin B.S., Bossaller L., De Nardo D., Ratter J.M., Stutz A., Engels G., Brenker C., Nordhoff M., Mirandola S.R., Al-Amoudi A. (2014). The adaptor ASC has extracellular and ’prionoid’ activities that propagate inflammation. Nat. Immunol..

[24] Kolly L., Karababa M., Joosten L.A.B., Narayan S., Salvi R., Petrilli V., Tschopp J., Berg W.B.V.D., So A.K.-L., Busso N. (2009). Inflammatory Role of ASC in Antigen-Induced Arthritis Is Independent of Caspase-1, NALP-3, and IPAF. J. Immunol..

[25] Protti M.P., De Monte L. (2020). Dual Role of Inflammasome Adaptor ASC in Cancer. Front. Cell Dev. Biol..

[26] Kitazawa M., Hida S., Fujii C., Taniguchi S., Ito K., Matsumura T., Okada N., Sakaizawa T., Kobayashi A., Takeoka M. (2017). ASC Induces Apoptosis via Activation of Caspase-9 by Enhancing Gap Junction-Mediated Intercellular Communication. PLoS ONE.

[27] Fink S.L., Cookson B.T. (2005). Apoptosis, Pyroptosis, and Necrosis: Mechanistic Description of Dead and Dying Eukaryotic Cells. Infect. Immun..

[28] Fernandes-Alnemri T., Wu J., Yu J.-W., Datta P., Miller B., Jankowski W., Rosenberg S., Zhang J., Alnemri E.S. (2007). The pyroptosome: A supramolecular assembly of ASC dimers mediating inflammatory cell death via caspase-1 activation. Cell Death Differ..

[29] Hsiao K., Chapman P., Nilsen S., Eckman C., Harigaya Y., Younkin S., Yang F., Cole G. (1996). Correlative Memory Deficits, A Elevation, and Amyloid Plaques in Transgenic Mice. Science.

[30] Kawarabayashi T., Younkin L.H., Saido T.C., Shoji M., Ashe K.H., Younkin S.G. (2001). Age-dependent changes in brain, CSF, and plasma amyloid (beta) protein in the Tg2576 transgenic mouse model of Alzheimer’s disease. J. Neurosci..

[31] Howlett D.R., Richardson J.C. (2009). The pathology of APP transgenic mice: A model of Alzheimer’s disease or simply overex-pression of APP?. Histol. Histopathol..

[32] Shamaa O.R., Mitra S., Gavrilin M.A., Wewers M.D. (2015). Monocyte Caspase-1 Is Released in a Stable, Active High Molecular Weight Complex Distinct from the Unstable Cell Lysate-Activated Caspase-1. PLoS ONE.

[33] Boucher D., Monteleone M., Coll R.C., Chen K.W., Ross C.M., Teo J.L., Gomez G.A., Holley C.L., Bierschenk D., Stacey K.J. (2018). Caspase-1 self-cleavage is an intrinsic mechanism to terminate inflammasome activity. J. Exp. Med..

[34] Van Groen T., Liu L., Ikonen S., Kadish I. (2003). Diffuse amyloid deposition, but not plaque number, is reduced in amyloid precursor protein/presenilin 1 double-transgenic mice by pathway lesions. Neuroscience.

[35] Yamaguchi H., Maat-Schieman M.L.C., Van Duinen S.G., Prins F.A., Neeskens P., Natté R., Roos R.A.C. (2000). Amyloid β Protein (Aβ) Starts to Deposit as Plasma Membrane-Bound Form in Diffuse Plaques of Brains from Hereditary Cerebral Hemorrhage with Amyloidosis-Dutch Type, Alzheimer Disease and Nondemented Aged Subjects. J. Neuropathol. Exp. Neurol..

[36] D’Andrea M.R., Cole G.M., Ard M.D. (2004). The microglial phagocytic role with specific plaque types in the Alzheimer disease brain. Neurobiol. Aging.

[37] Stalder M., Phinney A., Probst A., Sommer B., Staufenbiel M., Jucker M. (1999). Association of Microglia with Amyloid Plaques in Brains of APP23 Transgenic Mice. Am. J. Pathol..

[38] Koistinaho M., Ort M., Cimadevilla J.M., Vondrous R., Cordell B., Bures J., Higgins L.S. (2001). Specific spatial learning deficits become severe with age in -amyloid precursor protein transgenic mice that harbor diffuse -amyloid deposits but do not form plaques. Proc. Natl. Acad. Sci. USA.

[39] Condello C., Yuan P., Grutzendler J. (2018). Microglia-Mediated Neuroprotection, TREM2, and Alzheimer’s Disease: Evidence from Optical Imaging. Biol. Psychiatry.

[40] Schroder K., Tschopp J. (2010). The Inflammasomes. Cell.

[41] Ismael S., Wajidunnisa S.K., McDonald M.P., Liao F.-F., Ishrat T. (2021). ER stress associated TXNIP-NLRP3 inflammasome activation in hippocampus of human Alzheimer’s disease. Neurochem. Int..

[42] Kuri P., Schieber N.L., Thumberger T., Wittbrodt J., Schwab Y., Leptin M. (2017). Dynamics of in vivo ASC speck formation. J. Cell Biol..

[43] Bryan N.B., Dorfleutner A., Kramer S.J., Yun C., Rojanasakul Y., Stehlik C. (2010). Differential splicing of the apoptosis-associated speck like protein containing a caspase recruitment domain (ASC) regulates inflammasomes. J. Inflamm..

[44] Manich G., Cabezón I., Augé E., Pelegrí C., Vilaplana J. (2016). Periodic acid-Schiff granules in the brain of aged mice: From amyloid aggregates to degenerative structures containing neo-epitopes. Ageing Res. Rev..

[45] Sahillioğlu A.C., Ozören N. (2015). Artificial Loading of ASC Specks with Cytosolic Antigens. PLoS ONE.

[46] Iliff J.J., Lee H., Yu M., Feng T., Logan J., Nedergaard M., Benveniste H. (2013). Brain-wide pathway for waste clearance captured by contrast-enhanced MRI. J. Clin. Investig..

[47] Ba Kress B.T., Iliff J.J., Xia M., Wang M., Wei H.S., Zeppenfeld D., Xie L., Kang H., Xu Q., Liew J.A. (2014). Impairment of paravascular clearance pathways in the aging brain. Ann. Neurol..

[48] Baroja-Mazo A., Martín-Sánchez F., Gomez I.A., Martinez C.M., Amores-Iniesta J., Compan V., Barberà-Cremades M., Yagüe J., Ruiz-Ortiz E., Anton J. (2014). The NLRP3 inflammasome is released as a particulate danger signal that amplifies the inflammatory response. Nat. Immunol..

[49] Kulijewicz-Nawrot M., Verkhratsky A., Chvatal A., Syková E., Rodríguez J.J. (2012). Astrocytic cytoskeletal atrophy in the medial prefrontal cortex of a triple transgenic mouse model of Alzheimer’s disease. J. Anat..

[50] Rodríguez-Arellano J., Parpura V., Zorec R., Verkhratsky A. (2016). Astrocytes in physiological aging and Alzheimer’s disease. Neuroscience.

[51] Yassa A.M. (2014). Ground zero in Alzheimer’s disease. Nat. Neurosci..

[52] Shu S., Zhu H., Tang N., Chen W., Li X., Li H., Pei L., Liu D., Mu Y., Tian Q. (2016). Selective Degeneration of Entorhinal-CA1 Synapses in Alzheimer’s Disease via Activation of DAPK1. J. Neurosci..

[53] Capogna M. (2011). Neurogliaform cells and other interneurons of stratum lacunosum-moleculare gate entorhinal-hippocampal dialogue. J. Physiol..

[54] Carro E., Trejo J., Gomez-Isla T., Leroith D., Aleman I.T. (2002). Serum insulin-like growth factor I regulates brain amyloid-β levels. Nat. Med..

[55] Franklin K., Paxinos G. (2007). The Mouse Brain in Stereotaxic Coordinates.

